# Is Penile Doppler Ultrasonography Overdiagnosed?

**DOI:** 10.7759/cureus.36450

**Published:** 2023-03-21

**Authors:** Mehmet Y Salman, Serkan Dogan, Kenan Y Yildiz

**Affiliations:** 1 Urology, Sehit Prof. Dr. Ilhan Varank Training & Research Hospital, Istanbul, TUR; 2 Urology, Ankara Bilkent City Hospital, Ankara, TUR

**Keywords:** venous insufficiency, arterial insufficiency, vasculogenic pathology, indication, penile doppler ultrasound

## Abstract

Aim: The objective of this study was to determine whether penile Doppler ultrasonography (USG) examinations, combined with the administration of intracavernosal vasoactive agents, were correctly performed as a second-line diagnostic method with the proper indications in a select patient group.

Methods: A total of 200 patients who underwent penile Doppler ultrasounds in our clinic were included in the study. Patients’ demographic data were collected, information about their medical-sexual history was taken, physical examinations were performed, and laboratory analyses were conducted. Patients were informed in detail about the process, and their consent was given prior to the study. Procedure outcomes and complications were also recorded. The relationship of vasculogenic pathologies was analysed, and the rate of abnormal results and complications was compared with the existing literature.

Results: Following the investigations, abnormal results were found in 24.5% of patients, while this rate was 6.8% in patients under the age of 40. Arterial insufficiency was found in 31 patients and venous insufficiency in 18 patients. Five patients had both pathologic conditions. No statistically significant correlation was found between arterial insufficiency and age, while venous insufficiency was significantly correlated with age (p=0.006).

Conclusion: Penile Doppler ultrasounds should only be ordered for a carefully selected patient group. Ordering ultrasounds without a proper indication can result in unnecessary labour and financial loss, as well as an increase in invasive procedures.

## Introduction

Erectile dysfunction (ED) is the most common sexual problem, especially in older men. Although many studies have reported that the incidence and prevalence of ED increase with age, there are publications reporting that it is also commonly seen in young men [1-3]. The cases reported have varied significantly across studies. This has been attributed to various factors, such as questioning techniques, age distributions, and sociocultural differences in the populations studied [4]. Vascular problems, neurologic diseases, endocrinologic diseases, trauma, pharmacological agents, and psychological factors are involved in the etiology. There are two major etiological groups, namely organic causes and psychogenic causes [5-7]. To establish the etiology, the history of the patient, a physical examination, and first-line investigations are needed. Both medical and sexual histories should be reviewed in detail, and validated survey forms should be used [5].

A review of medical and sexual histories, a physical examination, and first-line investigations are sufficient to arrange treatment for the majority of patients [8,9]. The remaining patients, however, require advanced studies and analyses to be performed. One of these studies is penile Doppler ultrasonography (USG), which is frequently used in clinics in combination with injectable vasoactive agents [10]. It is a minimally invasive second-line diagnostic method used when ED cases are thought to stem from hemodynamic pathology of the penis. It is highly effective in detecting arterial insufficiency and/or venous leaks that are vasculogenic problems [11]. However, studies have increasingly found normal values in patients undergoing penile Doppler USG [12]. The majority of ED patients are diagnosed with psychogenic ED [13]. If minimally invasive, penile Doppler USG should only be performed on selected patients who request it, in order to avoid the possible complications, labour, and financial loss this procedure may incur. In our study, we aimed to determine whether this examination was performed more often than necessary.

## Materials and methods

Patients

During the study, a total of 864 patients with a pre-diagnosis of ED were examined in outpatient clinics. Penile Doppler USG was performed on a total of 248 patients. A total of 200 patients who came to our clinic with complaints of ED and who underwent penile Doppler USG between September 2016 and March 2018 were included in the study.

A total of 48 patients were excluded from the study who had undergone surgery on the penis, Peyronie’s disease, connective tissue disease, radical prostatectomy, received radiotherapy to the pelvic area, previously developed priapism, or who were found by the cardiology department to have a high risk (Princeton III Consensus). Patients’ demographic information was recorded. In addition, we recorded how thoroughly patients were questioned in the outpatient clinic, whether they filled out a validated scale, what medical treatment was recommended, and whether they were informed in detail about the procedure.

The medical and sexual histories of patients were received by the specialist who performed the procedure, and the patients were informed about the examination. Patients underwent penile Doppler USG, and the results were evaluated. The rate of vasculogenic problems and normal results were reviewed. The relationships between arterial insufficiency and venous leaks, which are a common vasculogenic problem associated with age, were statistically analysed.

The study was approved by the Sancaktepe Prof. Dr. Ilhan Varank Training and Research Hospital Scientific Research and Ethics Committee in September 2018 with the number 3208. All patients were informed in detail before the procedure, and written informed consent was given. The study was prepared under the Helsinki Declaration Criteria.

Penile Doppler USG procedure

All examinations were performed by two radiologists with at least eight years of experience in the profession. Before beginning, all patients were informed about the procedure and signed informed consent forms. All patients over the age of 40 and with a history of cardiovascular disease were referred to the cardiology department. The 7.5 MHz high-frequency superficial probe of the Mindray DC-7 ultrasound system device (Mindray Bio-Medical Electronics, Nanshan, Shenzhen, PRC) was used. The procedure was performed with patients in the supine position by tilting the penis towards the abdomen. The probe angle was between 300 and 600˚. Patients were injected with 30 mg of intracavernosal papaverine hydrochloride. As the vascular flow parameters, peak-systolic and end-diastolic flow rates were recorded at 1, 10, 20, and 30 minutes. Patients with a peak-systolic flow velocity <30 cm/s or end-diastolic flow velocity >3 cm/s at the 10-minute mark and who could not achieve their usual erection quality in the last month were administered a further 30 mg dose. Erection degrees were also recorded at 20 minutes as follows: 0 = no change, 1 = moderate tumescence, 2 = tumescence without rigidity, 3 = partial rigidity, 4 = rigid erection. A peak-systolic flow rate <30 cm/s was considered as arterial insufficiency, and an end-diastolic flow rate >3 cm/s was considered venous insufficiency.

Statistical analysis

Statistical analysis of the data obtained in this study was performed with SPSS 22.0 (Statistical Package for Social Sciences, IBM Corp., Armonk, NY) package software. Considering the prevalence of ED, taking the power of the study 80%, and accounting for a Type 1 error margin of 0.05, it was determined that at least 33 patients were required for each group.

Numeric mean and standard deviation were used for descriptive statistics. For normally distributed peak-systolic flow rates, Student’s t-tests were used to compare patients with and without arterial insufficiency according to age. Similarly, Student’s t-tests were used to compare the age of patients with and without venous insufficiency as a result of the end-diastolic flow rate. A p<0.05 value was considered statistically significant.

## Results

A total of 200 patients were included in the study. The mean peak-systolic flow rate was found to be 15.4 ± 4.4 cm/s in patients with arterial insufficiency and 49.1 ± 27.6 cm/s in patients without. The mean end-diastolic flow rate was found to be 6.3 ± 0.9 cm/s in patients with venous insufficiency and 1.6 ± 0.5 cm/s in patients without. The mean age of patients with arterial insufficiency was 57.7 ± 11.1 years, while the mean age of patients without arterial insufficiency was 54.6 ± 12.6 years. No significant difference was found between the patients with and without arterial insufficiency in terms of age (p=0.191). The mean age of patients with venous insufficiency was 62.61 ± 11.5 years, while the mean age of patients without venous insufficiency was 54.3 ± 12.3 years. There was a statistically significant difference between the patients with and without venous insufficiency in terms of age (p=0.006).

The average height of patients with arterial insufficiency was 168.9 ± 13.2 cm, while the average height of patients without arterial insufficiency was 171.2 ± 12.1 cm (p=0.274). The average height of patients with venous insufficiency was 170.3 ± 12.4 cm, while the average height of patients without venous insufficiency was 170.9 ± 13.1 cm (p=0.693). While the mean weight of patients with arterial insufficiency was 78.3 ± 14.3 kg, the average weight of patients without arterial insufficiency was 69.2 ± 12.6 kg (p=0.012). The mean weight of patients with venous insufficiency was 70.6 ± 11.4 kg, while the mean weight of patients without venous insufficiency was 70.2 ± 12.2 kg (p=0.352). The mean body mass index (BMI) was also found to be significantly higher in patients with arterial insufficiency (p=0.018) (Table 1).

**Table 1:** Comparison of demographic data between groups

	Arterial insufficiency positive	Arterial insufficiency negative	P value
Age (year)	57.7±11.1	54.6±12.6	0.191
Length (cm)	168.9±13.2	171.2±12.1	0.274
Weight (kg)	78.3±14.3	69.2±12.6	0.012
BMI (kg/m^2^)	27.4±4.2	23.7±3.9	0.018
	Venous insufficiency positive	Venous insufficiency negative	
Age (year)	62.61±11.5	54.3±12.3	0.006
Length (cm)	170.3±12.4	170.9±13.1	0.693
Weight (kg)	70.3 ±11.4	69.6 ±12.2	0.352
BMI (kg/m^2^)	24.3±3.6	23.8±4	0.261

Of the 200 patients included in the study, 72 (36%) had hypertension, 59 (27.5%) had diabetes mellitus, 68 (34%) had cardiovascular disease, 19 (9.5%) had thyroid disease, and 18 (9%) had neurological disease. Of all patients, 108 (54%) were smokers or had used cigarettes at some point in their life. In addition, 78 (39%) patients had lower urinary tract symptoms (LUTS), and 62 (31%) patients were receiving medical treatment for this reason (Table 2).

**Table 2:** Comorbidities of patients LUTS: Lower urinary tract symptoms

	Positive	Negative	Total
Hypertension (n)	72	128	200
Cardiovascular Disease (n)	68	132	200
Diabetes Mellitus (n)	59	141	200
Thyroid Disease (n)	19	181	200
Neurological Disease (n)	18	182	200
LUTS (n)	78	122	200
Smoking (n)	108	92	200

Sixteen patients had undergone transurethral resection or open prostatectomy due to benign prostate hyperplasia. The mean BMI of patients was calculated to be 26.73±7.52 kg/m². Of the patients, 84 (42%) were overweight and 48 (24%) were obese.

**Figure 1:**
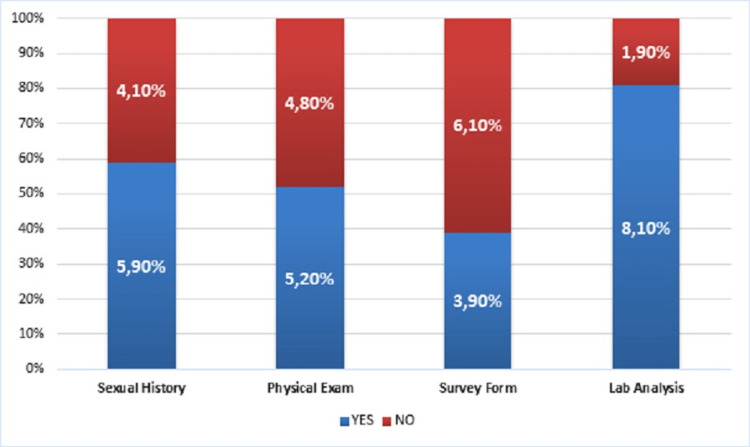
Rate of patients with sexual history requested, any survey form filled out, and physical examination and laboratory analysis performed.

Eighty-two (41%) patients reported that no detailed sexual history was requested in the outpatient clinic, 122 (61%) stated that they did not fill out any survey form, and 96 (48%) reported that they did not undergo physical examination. Laboratory analysis was ordered for 162 (81%) patients (Figure 1).

Lifestyle modification was recommended for 134 (67%) patients. Medical therapy was recommended before second-line investigations for 143 (71.5%) patients.

After the procedure, 13 (6.5%) patients developed complications. These complications ranged from Grade 1 to Grade 3a according to the Clavien-Dindo classification. Subcutaneous ecchymosis was found in five patients, pain in the application site in four patients, painful erection in four patients, priapism in three patients, ziness in two patients, and tachycardia in one patient. Only one patient required corporoglanular T-shunt application.

## Discussion

Penile Doppler USG is a highly effective examination in the diagnosis of vasculogenic ED. ED is especially prevalent in some diseases that cause vascular problems, such as diabetes mellitus, cardiovascular and peripheral artery diseases, metabolic syndromes, obesity, and hyperlipidemia [11-14]. This form of examination, which was used for the first time in combination with iatrogenic erection by vasoactive agents in 1985, is the most commonly used today [15]. Papaverine, prostaglandin, and phentolamine are the most frequently used agents for this purpose. Although there are studies using oral phosphodiesterase-5 inhibitors, problems with efficacy and an increased risk of priapism have been noted [16,17]. In our study, we used papaverine hydrochloride. There is a shortage of numerical data concerning the frequency of penile Doppler USG application. Generally, the studies are related to the prevalence of abnormal data obtained.

According to the investigation results, a peak-systolic flow rate >30 cm/s, an end-diastolic flow rate <3 cm/s, and a resistance index >0.8 are accepted as normal. These values are included in the European Association of Urology (EAU) guidelines, although there are also echoles and studies that accept different values [4,18]. In this study, we evaluated the findings according to EAU guidelines. When all patients were included, the rate of abnormal results was found to be 24.5%, while this rate was 6.8% in patients under 40 years of age. Studies have reported very different results for the rate of abnormal outcomes with penile Doppler USG examinations [19]. While this result is supported by the literature, it opens up a discussion on the indications of investigation. This is because, although there is a wide range of methods, only around 50% of abnormal results are seen [20-22]. Particularly, there are numerous studies reporting a higher rate of psychogenic impotence in young patients [12,13,18]. In our study, the rate of abnormal findings was very low in the population under 40 years of age. Obesity is also one of the factors that increase ED. In particular, obesity negatively affects peak-systolic flow velocity following penile Doppler USG examinations [23]. In our study, the BMI of patients with arterial insufficiency was significantly higher.

A comprehensive medical and sexual history, physical examination, and basic laboratory evaluation are the first steps in all guidelines for diagnosing and treating ED. After the introduction of phosphodiesterase-5 inhibitors, radical changes occurred in the diagnosis and treatment of ED. Achieving rigid erections with these drugs has begun to be evaluated in treating arterial insufficiency and, less commonly, patent veno-occlusive disease. As a result, the frequency of invasive examinations has decreased. Invasive assessments should be performed as a second-line diagnostic method and in carefully selected patient groups. Unfortunately, according to the feedback we received from patients, a detailed history was requested in 60% of cases, while physical examinations were neglected in approximately 50% of patients. In comparing our results with other studies, the detection rate of vascular pathologies generally seems to be lower. This indicates that adequate care was not taken in selecting patients. The rate of patients under 40 years old was 22%, and abnormal results were found in only three of these patients. This result is consistent with the finding that, especially in younger patients, psychogenic impotence constitutes the larger part of ED [12,18]. However, it is clear that the rate of patients requiring penile Doppler USG will significantly decrease if proper questioning, physical examination, and laboratory analysis are performed.

Although it is a minimally invasive imaging technique, side effects such as tachycardia, dizziness, and headaches are seen more commonly, especially in patients with venous leaks, due to the large volume of vasoactive agents entering the circulatory system [24]. In addition, complications ranging from painful or pain-free prolonged erections to priapism may also be experienced. Local complications, such as pain and ecchymosis in the site of application, and infrequent side effects, such as dyspeptic complaints, headaches, tachycardia, dizziness, and blurry vision, rarely require medical treatment [24]. On the other hand, priapism is a serious complication that can result in the need for an intracavernosal sympathomimetic injection, aspiration, shunts, and even surgery [24,25]. The rate of complication has been reported as about 7-12% in different studies [18,24]. In our study, the rate of complication was found to be 6.5%. Eight of the 13 patients had a normal penile Doppler USG result.

Study limitations

The main limitation of the study is the relatively small number of patients and being conducted in a single center. In addition we could not include a control group and the study was designed as a retrospective study.

## Conclusions

We think that better evaluation of ED patients in urology outpatient clinics will prevent unnecessary penile Doppler USG examinations from being ordered without an exact indication. In conclusion, the financial burden will be reduced, and complications related to performing this procedure without indications will be avoided. If there are no contraindications, a reassessment should be made after using phosphodiesterase-5 inhibitors. Both ethically and medicolegally, we believe that all invasive examinations, even if minimally invasive, should only be ordered when exact indications are present.
